# Long-Chain acyl-CoA Synthetase LACS2 Contributes to Submergence Tolerance by Modulating Cuticle Permeability in Arabidopsis

**DOI:** 10.3390/plants9020262

**Published:** 2020-02-18

**Authors:** Li-Juan Xie, Wei-Juan Tan, Yi-Cong Yang, Yi-Fang Tan, Ying Zhou, De-Mian Zhou, Shi Xiao, Qin-Fang Chen

**Affiliations:** State Key Laboratory of Biocontrol, Guangdong Provincial Key Laboratory of Plant Resources, School of Life Sciences, Sun Yat-sen University, Guangzhou 510275, China; xielij3@mail.sysu.edu.cn (L.-J.X.); twjtwjtwjok@yeah.net (W.-J.T.); yangyic@mail2.sysu.edu.cn (Y.-C.Y.); tanyf9@mail2.sysu.edu.cn (Y.-F.T.); zhouyingwp@163.com (Y.Z.); demychow@163.com (D.-M.Z.); xiaoshi3@mail.sysu.edu.cn (S.X.)

**Keywords:** *Arabidopsis thaliana*, cuticle, LONG-CHAIN ACYL-COA SYNTHETASE, permeability, submergence

## Abstract

In *Arabidopsis thaliana*, LONG-CHAIN ACYL-COA SYNTHETASEs (LACSs) catalyze the synthesis of long-chain acyl-CoAs and function in diverse biological processes. We have recently revealed that LACS2 is primarily involved in the production of polyunsaturated linolenoyl-CoA, essential for the activation of ethylene response transcription factors-mediated hypoxia signaling. Here, we further reported the dual role of LACS2 in the regulation of submergence tolerance by modulating cuticle permeability in Arabidopsis cells. *LACS2*-overexpressors (*LACS2-OEs*) showed improved tolerance to submergence, with higher accumulation of cuticular wax and cutin in their rosettes. In contrast, knockout of *LACS2* in the *lacs2-3* mutant resulted in hypersensitivity to submergence with reduced wax crystals and thinner cutin layer. By analyses of plant surface permeability, we observed that the hypoxic sensitivities in the *LACS2-OEs* and *lacs2-3* mutant were physiologically correlated with chlorophyll leaching, water loss rates, ionic leakage, and gas exchange. Thus, our findings suggest the role of LACS2 in plant response to submergence by modulating cuticle permeability in plant cells.

## 1. Introduction

Submergence-induced hypoxia is one of the most important abiotic stresses, which seriously affects plant growth, development, and yields [1]. To cope with the insufficient oxygen supply under long-term submergence conditions, plants have developed a series of adaptations at cellular, tissue, and organismal levels [1]. These processes are tightly under the regulation of multiple signaling networks and their interaction [2,3,4,5]. In particular, the N-end rule-controlled subgroup VII ETHYLENE-RESPONSE FACTOR (ERF-VII), is considered to be a key regulator for oxygen sensing in plant cells [6,7].

In the plant, the ERF-VII members, such as RELATED TO APETALA 2.12 (RAP2.12), are subcellularly localized to the plasma membrane by interacting with membrane-associated ACYL-COA BINDING PROTEIN 1 (ACBP1) and ACBP2 under normoxic conditions. Upon oxygen deprivation, RAP2.12 is released from the ACBP1–ERF-VII complex and translocated into the nucleus to activate the expression of hypoxia-responsive genes. Further, upon reoxygenation, the levels of ERF-VII proteins are controlled by ubiquitination and degradation though the N-end rule proteolysis pathway [1,4]. More recently, two independent studies reveal that under hypoxia, lipid signals promote the dissociation of RAP2.12 from ACBPs [7,8]. Specifically, Schmidt et al. (2019) revealed that higher C18:1-CoA to C16:0-CoA ratio induced by hypoxia, triggers the release of transcription factor RAP2.12 from its interaction partner ACBP1 at the plasma membrane [7]. Moreover, our results suggested that polyunsaturated long-chain acyl-CoAs are predominantly involved in the activation of plant hypoxia signaling by modulating ACBP–ERF-VII dynamics [8]. These findings imply that the polyunsaturation of long-chain acyl-CoAs is likely an important mechanism in the regulation of plant hypoxia sensing.

Synthesis of long-chain acyl-CoAs from free fatty acids is catalyzed by the long-chain acyl-CoA synthetases (LACSs) in plant cells [9]. In Arabidopsis, there are nine genes encoding LACS proteins with various roles in lipid metabolism [9]. Among them, LACS1 and LACS2 are mainly involved in the synthesis of cutin and cuticular waxes, whereas LACS6 and LACS7 function in providing fatty acids for β-oxidation [10,11,12,13,14]. Besides, both LACS1 and LACS9 contribute to synthesis of acyl-CoAs for triacylglycerol (TAG) accumulation [15]. Recently, the functions of these LACSs enzymes in plant development and stress responses have been widely investigated by reverse genetics and biochemical approaches. Analysis of *lacs1 lacs4* double mutants show that both LACS1 and LACS4 are required for pollen coat formation [16]. In particular, Tang et al. (2007) revealed that the mutation of LACS2 in Arabidopsis improves plant tolerance to *Botrytis cinerea* but confers susceptibility to avirulent *Pseudomonas syringae*, suggesting that LACS2-mediated plant cutin or cuticle structure may play an indispensable role in plant tolerance to environmental stress [17].

We have recently suggested that LACS2 is involved in submergence tolerance through modulating the translocation of ERF-VII transcription factor from the membrane to the nucleus. It is also conceivable that upon submergence, the altered expression of LACS2 may affect the integrity and permeability of the plant cuticle, leading to the accumulation of water in the apoplastic space and resulting in physiological disorders. These alterations are likely functional in hypoxia signaling by affecting the gas exchanges of plant cells, especially under long-term submergence conditions. In this study, by analyzing the loss-of-function (*lacs2-3*) and overexpression (*LACS2-OEs*) lines of *LACS2*, we investigated the changes of the wax crystals and cuticle layer, the cuticular wax and cutin composition, and the cuticle permeability upon submergence exposure. Our findings suggest that the sensitivities of *lacs2-3* mutant and *LACS-OE* lines to submergence are directly associated with their surface permeabilities.

## 2. Results

### 2.1. LACS2 Is Involved in Maintenance of Submergence-Induced Alterations of Epicuticular Wax and Cutin

**Figure 1:**
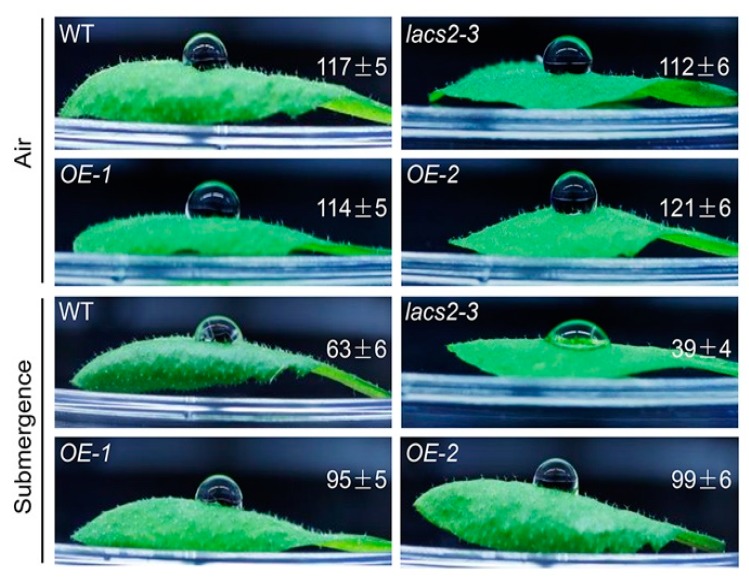
Wild type (WT), *lacs2-3*, and *LACS2-OEs* leaves showed changed hydrophobicities upon submergence. The hydrophobic cuticular wax of WT, *lacs2-3*, and *LACS2-OE* lines were detected by water droplets under normal growth (Air) and submergence (Submergence) conditions. Hydrophobicity of the leaf cuticular wax was assessed using the contact angle of small droplets of water, and contact angles <90° indicate a hydrophilic surface, whereas those of >90° indicate a hydrophobic surface. The experiments have been biologically repeated three times and similar results were obtained. For each experiment, at least six leaves were used per genotype. Data are average values ± SD (*n* = 3) calculated from three independent experiments.

Our previous microarray data showed that submergence substantially suppresses the transcripts of several genes involved in cuticular lipid metabolism [18]. Moreover, the Arabidopsis LACS2 plays a vital role in cuticle development [11]. To investigate the potential association of LACS2-mediated cuticular lipid biosynthesis with submergence, we first tested the hydrophobicity of the leaf cuticle in response to submergence treatment. A decrease of the cuticular wax load under hypoxic stress may lead to the loss of leaf surface hydrophobicity [19]. To this end, the leaves of 4-week-old wild type, *lacs2-3*, and *LACS2-OEs* (*OE-1* and *OE-2*) plants were harvested before and after 1-day submergence, and a water droplet was placed on the adaxial side of the leaf. As shown in Figure 1, no visible difference was observed in the contact angles among the leaves of the wild type, *lacs2-3*, and *LACS2-OEs* plants under normal growth conditions. However, after 1 d of submergence, the *lacs2-3* plants became more hydrophilic than the wild type leaves, as the contact angle of the *lacs2-3* plants dropped to 39 ± 4°. By contrast, the contact angles of the *LACS2-OE* lines were significantly larger than that of the wild type after submergence (Figure 1). These findings suggest that the sensitivities of *lacs2-3* mutant and *LACS2-OE* lines to submergence may due to their changes in cuticular lipid metabolism.

**Figure 2:**
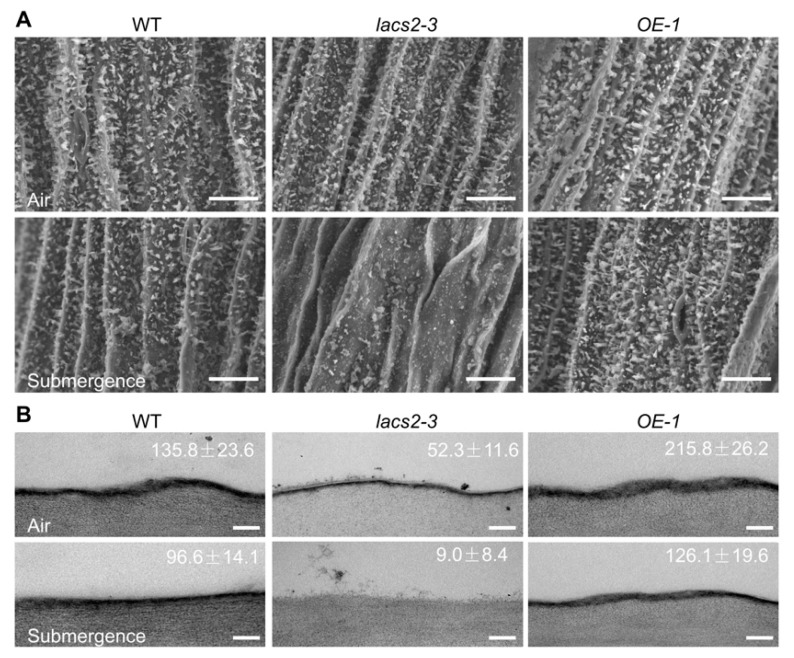
Alteration of cuticular wax and cutin loads on the stems of wild type (WT), *lacs2-3*, and *LACS2-OE* (*OE-1*) in response to submergence. (**A**) Scanning electron micrographs showing epicuticular wax crystals on the stems of six-week-old WT, *lacs2-3*, and *OE-1* under normal growth conditions (air) or submergence for three days (submergence). Scale bar, 10 μm. (**B**) Transmission electron microscopy images showing cuticle layers in the stems of six-week-old WT, *lacs2-3*, and *OE-1* under normal growth conditions (air) or submergence for three days (submergence). Scale bar, 500 nm. For each experiment, at least six leaves were used per genotype. Data are average values ± SD (*n* = 3) calculated from three independent experiments. The experiments have been biologically repeated three times and similar results were obtained.

To further investigate the effects of submergence on the biosynthesis and deposition of cuticular wax and cutin monomers, we observed the structures of epicuticular wax and the cutin layer of wild type, *lacs2-3*, and *OE-1* distal stems by scanning electron microscopy (SEM) or transmission electron microscopy (TEM). Under normal growth conditions, the wax crystal patterns on the distal stem surfaces of the *lacs2-3* mutant were similar to that of the wild type, whereas the *OE-1* distal stem surfaces showed a slightly higher density than the wild type (Figure 2A, upper panel). After submergence for three days, the wax crystals were substantially reduced on the wild type stem surfaces (Figure 2A, lower panel) and the wax deposition on *lacs2-3* stems almost completely disappeared, but the crystal densities on the *OE-1* stems remained almost unchanged compared to the wild type plants (Figure 2A, lower panel). In contrast to the changes of wax crystals, the cutin layer was thinner in the *lacs2-3* mutant but thicker in the *OE-1* stems compared to the wild type under either normal conditions or after three days of submergence (Figure 2B).

### 2.2. Cuticular Wax and Cutin Polyester Monomer Profiles of Wild type (WT), lacs2-3, and LACS2-OEs Leaves upon Submergence Exposure

**Figure 3:**
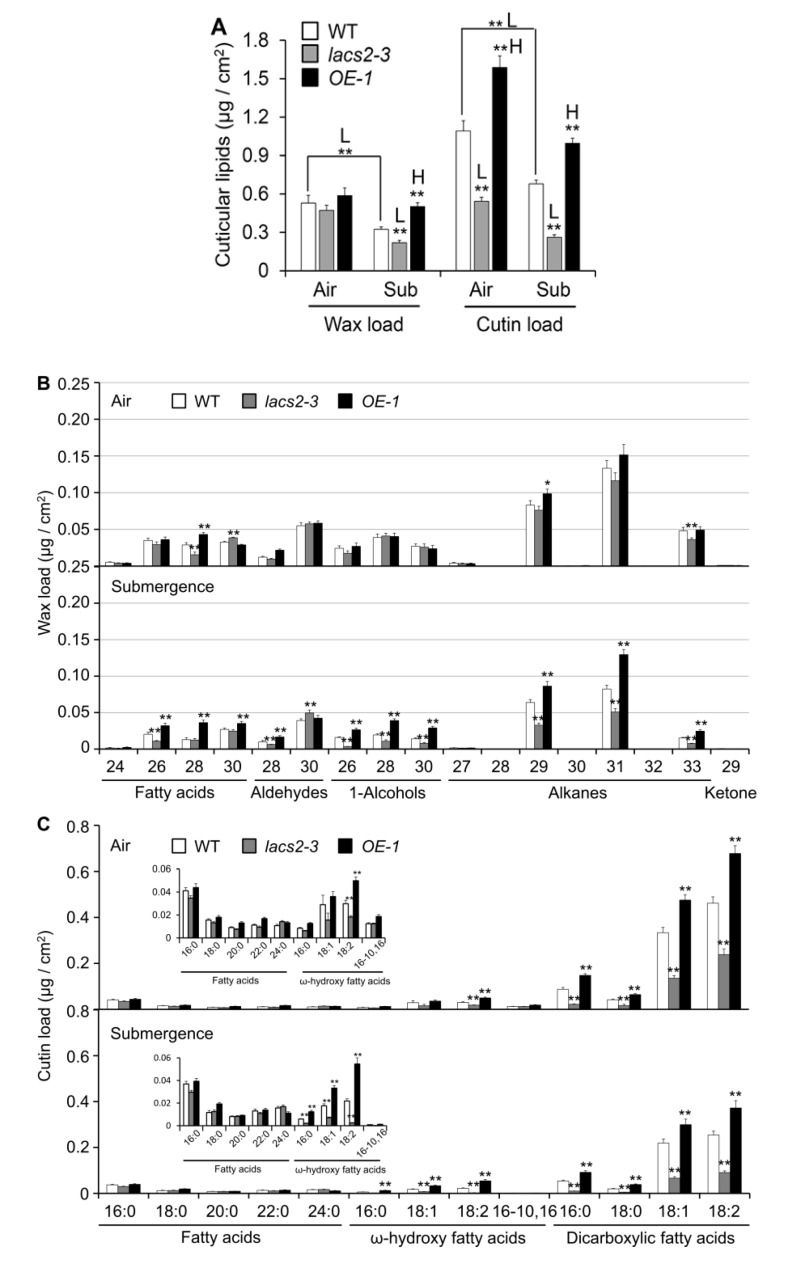
Cuticular wax and cutin Polyester monomer profiles of wild type (WT), *lacs2-3*, and *LACS2-OEs* leaves upon submergence exposure. (**A**) The levels of total amounts of wax and cutin loads of WT, *lacs2-3*, and *LACS2-OE* (*OE-1*) leaves under normal growth (air) and submergence (submergence) conditions. (**B**) and (**C**) The levels of different molecular species of wax (**B**) and cutin loads (**C**) of WT, *lacs2-3*, and *LACS2-OE* (*OE-1*) leaves under normal growth (Air) and submergence (Submergence) conditions. Leaves of four-week-old WT, *lacs2-3*, and *OE-1* were collected before submergence (air) and after three-day submergence treatment (submergence). Wax (**B**) and cutin (**C**) coverage is expressed as μg/cm^2^ leaf surface area. Contents of different molecular species of fatty acids and ω-hydroxy fatty acids are shown at the top left (**C**). For each experiment, at least six plants (technical replicates) were analyzed per genotype. Asterisks with “H” or “L” indicate significantly higher or lower level than that of WT (* *P* < 0.05, ** *P* < 0.01 by Student’s *t*-test).

To elucidate the role of LACS2 in submergence-induced wax and cutin biosynthesis, four-week-old wild type, *lacs2-3*, and *OE-1* plants were untreated or submerged for three days and their leaves were collected for wax and cutin profiling. As shown in Figure 3A, the levels of total wax were similar in the wild type, *lacs2-3*, and *OE-1* leaves under normal conditions. However, the total wax amounts declined dramatically in all genotypes after submergence. In particular, the wax load was significantly lower and higher in the *lacs2-3* and *OE-1* line, respectively, than in the wild type after submergence (Figure 3A). More specifically, the amounts of C26 fatty acid, C28 aldehyde, C26, C28, and C30 primary alcohols, and C29, C31, and C33 alkanes of cuticular wax were significantly lower in the *lacs2-3* mutant, but higher in the *OE-1* line than in the wild type (Figure 3B). Consistent with the TEM data, the levels of cutin significantly declined in the *lacs2-3* mutant but accumulated significantly in the *OE-1* line compared to that of the wild type under both normal and submergence conditions (Figure 3A). In particular, 18:1 and 18:2 ω-hydroxy fatty acids, and 16:0, 18:0, 18:1, and 18:2 dicarboxylic fatty acids of cutin monomers declined substantially in the *lacs2-3* mutant, but were maintained at higher levels in the *OE-1* line compared to the wild type after submergence (Figure 3C). Thus, these results reveal that submergence severely affects the deposition of cuticular wax and cutin monomers, which requires the function of LACS2.

### 2.3. Plant Surface Permeability Is Associated with Hyperhydricity and Hypoxia Signaling during Submergence

**Figure 4:**
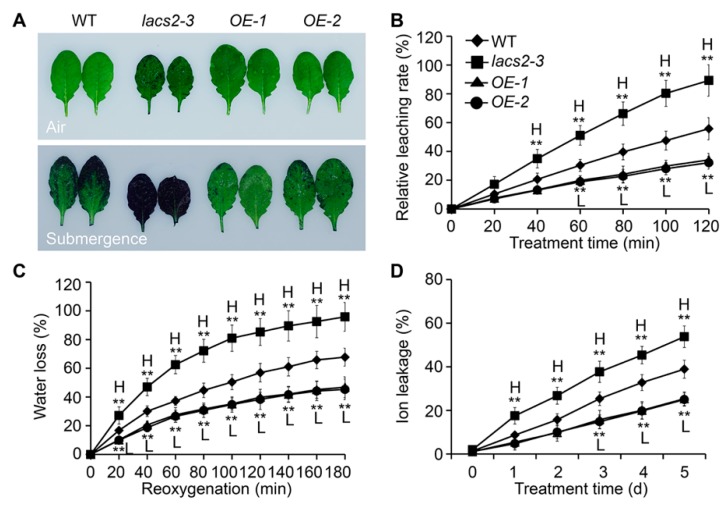
LACS2 regulated cuticle permeability in response to submergence treatment. (**A**) Cuticle permeability analysis in the leaves of four-week-old WT, *lacs2-3*, and *LACS-OEs* under normal growth (air) and submergence (submergence) conditions. The leaves were collected before and three days after treatment and stained with 0.05% toluidine blue. (**B**, **C** and **D**) Relative rates of chlorophyll leaching (**B**), water loss (**C**), and ion leakage (**D**) showing the cuticle permeability of four-week-old wild type (WT), *lacs2-3*, and *LACS2-OEs* leaves after submergence treatment for the indicated times. All of the experiments were performed for three times with similar results. The data are means ± SD (*n* = 6 technical replicates). Asterisks indicate significant differences from WT (** *P* < 0.01 by Student’s *t*-test). “H” and “L” indicate values that are significantly higher or lower, respectively, compared to that of WT.

The submergence-induced alterations of cuticular wax and cutin polyester may affect the permeability of the plant surface. To test this, we assessed the plant cuticle permeability of wild type, *lacs2-3*, and *OE-1* leaves by toluidine blue staining. Under normal conditions, the *lacs2-3* mutant showed a slightly elevated permeability compared to the wild type (Figure 4A, upper image), which is consistent with previous observations [11]. After the three-day submergence treatment, the *lacs2-3* leaves displayed much higher permeability than that of the wild type, as indicated by the increased staining densities (Figure 4A, bottom image). In contrast, the staining densities of the *LACS2-OE* leaves were remarkably lower than that of the wild type (Figure 4A, bottom image).

To further investigate the effects of altered cuticular wax and cutin structures on the plant hypoxia response under submergence, we measured the relative chlorophyll leaching and water loss rates of the wild type, *lacs2-3*, and *LACS2-OE* (*OE-1* and *OE-2*) plants before and after submergence. Compared to the wild type, chlorophyll leaching became more rapid in the *lacs2-3* leaves but was slower in the *OE-1* and *OE-2* leaves at 60 to 120 min after submergence (Figure 4B). During the de-submergence recovery (re-oxygenation) phase from 20 to 180 min, water loss was significantly enhanced in the *lacs2-3* mutant, and significantly reduced in the *OE-1* and *OE-2* lines, compared to that of the wild type (Figure 4C). Particularly, upon submergence, the ionic leakage percentages in the *lacs2-3* mutant were significantly higher than in the wild type plants and vice versa in the *LACS2-OE* lines (Figure 4D).

**Figure 5:**
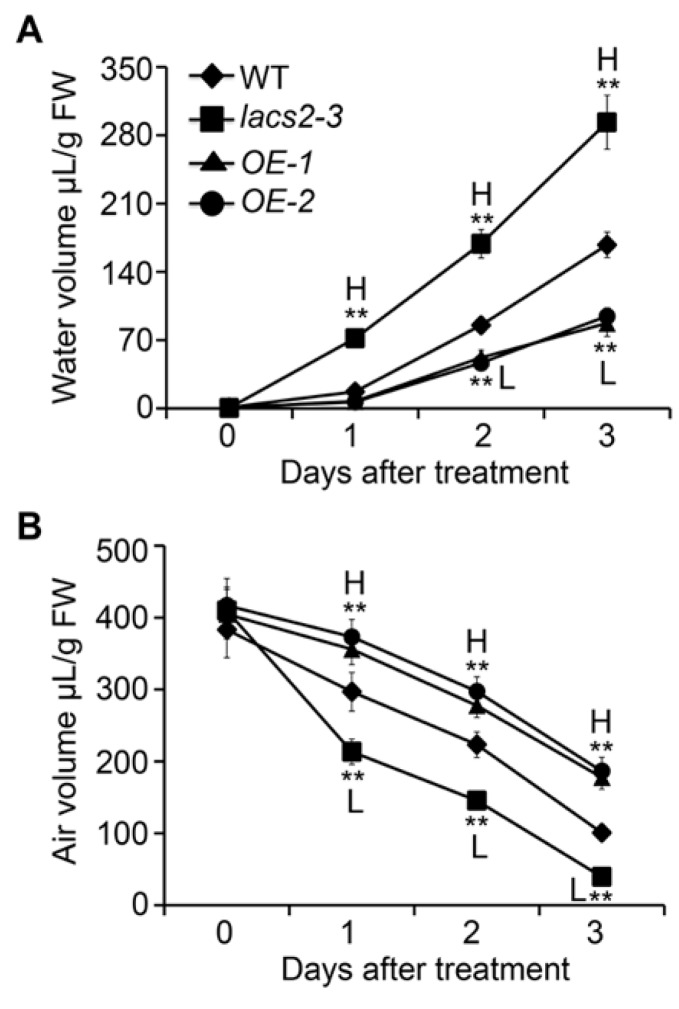
Altered expression of *LACS2* leads to apoplastic air availability in plant cells. Apoplastic water (**A**) and air volumes (**B**) of WT, *lacs2-3*, and *LACS2-OEs* before treatment and after submergence treatment for 1, 2, and 3 days. All of the experiments were performed for three times with similar results. The data are means ± SD (*n* = 6 technical replicates). Asterisks indicate significant differences from WT (** *P* < 0.01 by Student’s *t*-test). “H” and “L” indicate values that are significantly higher or lower, respectively, compared to that of WT.

Given that the alteration of cuticle permeability may result in excessive water in the plant cells and lead to physiological disorders [20], we further measured the volumes of water and air in the apoplastic space in the wild type, *lacs2-3*, and *LACS2-OE* plants at various times during submergence. In the wild type, the water volume increased significantly during submergence (Figure 5A). However, the water level was further elevated in the *lacs2-3* mutant but decreased in the *LACS2-OE* lines compared to the wild type from one to three days of submergence (Figure 5A). In contrast, compared with the wild type, the air volume declined drastically in the *lacs2-3* mutant, but remained at relative higher levels in the *OE-1* and *OE-2* lines under submergence (Figure 5B). These data suggest that LACS2 is a positive regulator of cuticle permeability under submergence stress.

## 3. Discussion

We have previously demonstrated that Arabidopsis LACS2 plays an essential role in hypoxia signaling by modulating long-chain acyl-CoA pools and ERF-VII–ACBP dynamics [8]. In this report, we present several lines of evidence to support the dual function of LACS2 in the modulation of cuticle permeability in plant response to submergence. First, deletion of Arabidopsis LACS2 resulted in reduced wax crystals and thin cutin layer, attenuated plant resistance to submergence stress (Figure 1 and Figure 2). Second, submergence triggered a significant decrease of wax and cutin compounds in rosettes, whose levels remarkably declined or increased in *lacs2-3* mutant and *LACS2-OE*, respectively, upon submergence exposure (Figure 3). Third, the *lacs2-3* mutant showed impaired biosynthesis of cuticular wax and cutin polyester, leading to higher chlorophyll leaching, water loss rates, ionic leakage, subsequently affecting the permeability of the plant surface and physiological disorders (Figure 4). Therefore, together with our previous findings [8], we suggest that LACS2 plays dual roles in plant response to hypoxia through modulating both ERF-VII signaling and cuticle-mediated membrane structure.

The plant cuticle is a continuous lipophilic layer composed of cuticular waxes and cutin polyesters. The plant cuticle covers the surface of all aerial organs and is the first physical barrier of defense against pathogens or abiotic stresses [21,22,23,24,25]. Wax is composed of very-long-chain (VLC) fatty acids and their derivatives and cutin is a structural polymer cross-linked with C16 and C18 fatty acids and glycerols. The production of C16 and C18 fatty acyl-CoA esters by LACS is the key step in the biosynthesis of wax and cutin [26]. As an essential component in cutin biosynthesis, Arabidopsis LACS2 plays a key role in the production of ω-hydroxy fatty acyl-CoA intermediates [11]. Furthermore, Arabidopsis ACBP1 and ACBP3 participate in cuticle formation in stems and leaves by their ability to shuttle very-long-chain acyl-CoAs (VLCACoAs) to elongases, and affecting the subsequent cuticular lipid synthesis [18,27,28,29].

Our data showing the significant decreases of wax and cutin loads in plants in response to submergence stress (Figure 3) is consistent with previous findings demonstrating the downregulation of genes involved in the biosynthesis and transport of cutin and wax [18], as well as cuticular lipid data using hypoxia as a stress inducer [30]. Given that the cuticle is the first barrier for gas exchange, particularly when the stomata are tightly closed under complete submergence conditions, the submergence-induced decreases of cuticular lipids may allow the formation of a permeable cuticle layer to take up water-soluble oxygen. Indeed, several previous findings suggest that carbon dioxide (CO_2_) and O_2_ can directly pass through the cuticle of plants when the stomata are closed [31,32]. In the terrestrial species *Rumex palustris*, the submergence-acclimated young leaves have a thinner cuticle that contributes to more influx of CO_2_ and O_2_ and stimulates photosynthesis, compared to the non-acclimated leaves [33]. More interestingly, in amphibious plants, the aquatic leaves showed lower cuticle thickness but higher gas permeability than the aerial leaves [32], suggesting that this may represent an evolutionarily adaptive strategy for plants to survive in response to different concentrations of oxygen, especially under submergence/flooding conditions. By measurements of air and water volumes in the apoplasts of submerged plants, we confirmed that the accumulation of water in the apoplasts significantly reduced the apoplastic air spaces, and this response was enhanced in the *lacs2-3* mutant, but attenuated in the *LACS2-OE* lines, especially after 2 and 3 days of submergence (Figure 4). These findings suggest that upon long-term submergence, cuticular permeability is tightly associated with the development of tissue hyperhydricity, which is hypothesized to impair gas exchange by the symplast and enhance the hypoxia response by activating the expression of hypoxia-responsive genes [20]. Meanwhile, previous studies revealed that the altered composition and permeability of the cuticle in *lacs2* single mutant or *lacs1 lacs2* double mutant changed plant tolerance to drought stress [14,34], further confirming the crucial function of cuticle permeability in modulating water loss in plants against variable environmental stresses.

**Figure 6:**
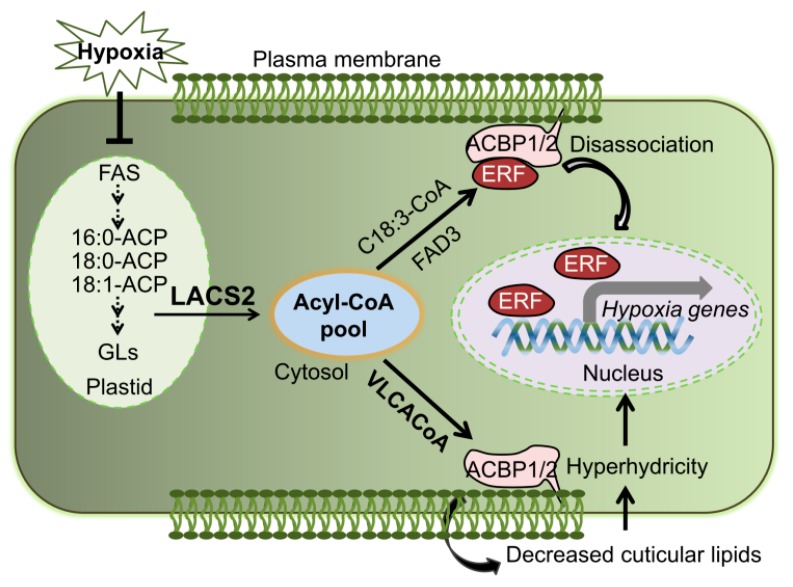
Model of the role of LACS2 in plant responses to hypoxia stress. Lipid remodeling is vitally necessary for hypoxic tolerance in plant [7,8,18,35]. By inactivation of fatty acid synthetase (FAS) and accelerating the fatty acid degradation, submergence-induced hypoxia decreases the fatty acid levels. LACS2 is essential for plant hypoxic tolerance and acyl-CoA metabolism during hypoxia. Specifically, the hypoxia-induced 18:3-CoA catalyzed by LACS2 and FAD3, interacts with ACBP1 or ACBP2, leads to the dissociation of the ACBPs–ERF-VII complex and subsequently activates the signaling cascades of ACBPs–ERF-VII. On the other hand, VLCACoAs produced by LACS2 are shuttled by ACBP1 or ACBP2 to facilitate the biosynthesis of cuticular lipids, which may contribute to plant surface permeability, cellular hyperhydrivity, and gas exchange under submergence conditions. ACBP; acyl-CoA-binding protein; ACP, acyl carrier protein; ERF, ethylene response factor; FAS, fatty acid synthase; VLCACoA, very-long-chain acyl-CoA.

Lipid remodeling is vitally necessary for hypoxic tolerance in plant [7,8,18,35]. Based on the results reported herein and in our previous study [8], we proposed that LACS2 is a primary regulator in plant response to submergence tolerance (Figure 6). By the inactivation of fatty acid synthetase (FAS) and accelerating the fatty acid degradation, submergence-induced hypoxia decreases the fatty acid levels. LACS2 is essential for plant hypoxic tolerance and acyl-CoA metabolism during hypoxia. Specifically, the hypoxia-induced 18:3-CoA catalyzed by LACS2 and FAD3, interacts with ACBP1 or ACBP2, leads to the dissociation of the ACBPs–ERF-VII complex and subsequently activates the signaling cascades of ACBPs–ERF-VII. On the other hand, long-chain acyl-CoAs (LCACoAs) or VLCACoAs produced by LACS2 are shuttled by ACBP1 or ACBP2 to facilitate the biosynthesis of cuticular wax and cutin monomers under normoxic conditions. Given that hypoxia stress, particularly that caused by submergence, suppresses the expression of all *LACS* genes [8], the lack of *LACS2* causes a significant reduction of wax and cutin polyesters to enhance cuticular permeability and the hypoxia response (Figure 4). Furthermore, the increased cuticle permeability causes an influx of water into the apoplasts of plant cells and elevates water loss when the water subsides, both of which may severely hamper plant survival under such environments.

## 4. Materials and Methods

### 4.1. Plant Materials, Culture Conditions, and Treatments

Wild type Arabidopsis used in this study was Columbia ecotype (Col-0). Characterizations of the mutant *lacs2-3* (CS65776), and *LACS2-OEs* (*OE-1* and *OE-2*) line (driven by the *UBQ10* promoter) have been previously described [8]. Arabidopsis seeds were surface-sterilized with 20% bleach containing 0.1% Tween 20 for 20 min, washed with distilled water five times, then sown on solid Murashige and Skoog (MS) medium (Sigma-Aldrich, St. Louis, MO, USA) plus with 1% sucrose. After incubated at 4 °C for 3 days, the plates were transferred to a growth room under a 16-h light/8-h dark (22 °C) photoperiod for 7 days. The seedlings were transplanted to soil and grown under the same conditions.

The submergence treatment was performed as described previously [18]. Briefly, four-week-old plants were submerged at depths of 10 cm beneath the water surface in the same growth room and harvested at the indicated times.

### 4.2. Scanning Electron Microscopy and Transmission Electron Microscopy

Scanning electron and transmission electron microscopy observations were performed as previously described [28,36]. For epicuticular wax crystal observation, distal stem segments from 6-week-old plants were removed and immersed in 0.1 M phosphate buffer (pH 7.0). The samples were then fixed by 1% osmium tetroxide for 4 h followed by air drying for 3 days. Dehydrated samples were mounted onto standard aluminum stubs and then coated with 30 nm of gold using a sputter coater. The images were viewed with a Hitachi S3400N scanning electron microscope.

For cuticle structure observation, distal stem segments from 6-week-old plants were taken and fixed in 0.1 M phosphate buffer (pH 7.4) containing 2.5% glutaraldehyde and 4% paraformaldehyde. After washing with phosphate buffer, samples were further fixed in 1% osmium tetroxide for 4 h at 4 °C. Next, samples were dehydrated, coated in Spurr’s resin, cut into thin layers, and put onto nickel grids. The layers were finally stained with uranyl acetate and lead citrate and observed with a JEM-2010 transmission electron microscope. The thickness of the cuticle layers (nm) was calculated using Image J software.

### 4.3. Leaf Hydrophobicity Analyses

Hydrophobicity of the leaf cuticle was tested by measuring the contact angle of water droplets on the leaves [19,37]. Briefly, leaves of four-week-old plants were collected before and after a 1-day submergence treatment. Then, 1 μL of water was dropped on the adaxial side of the leaf. The contact angle was measured using a contact angle meter (OCA15EC, Wavetest, Filderstadt, Germany).

### 4.4. Wax and Cutin Profiling

Wax profiling was carried out as described previously [38]. Briefly, fresh or frozen samples (100 mg) were immersed in chloroform for 30 s to extract the wax, and 20–50 µL (10 mg/50 mL) of n-Tetracosane (Sigma) was added as an internal standard. The extracted solution was dried with nitrogen and derived with 20 μL pyridine and 20 μL *N*,*O*-bis(trimethylsilyl) fluoroacetamide (BSTFA) (40 min at 70 °C). The derivate samples were then analyzed using gas chromatography–mass spectrometry (GC-MS).

For cutin analysis, samples were immersed in chloroform and methanol (1:1, *v*/*v*) in a glass vial for 2 weeks to remove phospholipids. Samples were then air-dried and reacted with methanol/HCl at 80 °C for 2 h. Saturated NaCl was used to end the reaction. Cutin was extracted with hexane, the extracted solution was dried with nitrogen, and the samples were derived with 20 μL pyridine and 20 μL BSTFA (40 min at 70 °C). Then, 20–50 μL (10 mg/50 mL) of dotriacontane (Sigma) was added to each sample as an internal standard. The derivate samples were then analyzed using GC-MS.

### 4.5. Analysis of Cuticle Permeability

The toluidine blue staining assay was performed as described previously [39]. Briefly, leaves of four-week-old plants were collected and immersed in 0.05% toluidine blue for 3 min. Leaves were then washed and photographed. For measurement of the chlorophyll leaching rate, whole plants were removed, weighed, and immersed in 80% ethanol. Absorbance of samples at different time points was determined at 664 nm and 647 nm. The chlorophyll leaching rates at individual time points were represented as percentages of that at 24 h after the initial immersion in 80% ethanol.

### 4.6. Measurement of Electrolyte Leakage and Water Loss

The electrolyte leakage assay was performed as previously described [40,41]. For calculation of the water loss rate, whole plants were collected after submergence in the light for 3 days and placed on plates under normal air conditions for the indicated time points. Weights were subsequently recorded and the water loss was represented by calculating the relative loss of fresh weight (%).

### 4.7. Measurement of Apoplastic Water and Air Volumes and Determination of Malate Dehydrogenase Activity

The volumes of apoplastic water and air were measured as described previously [20,41,42,43]. For calculation of the apoplastic water volume, whole plants submerged for the indicated times were collected, weighed, and centrifuged at 3000× *g* for 20 min at 4 °C. The plants were reweighed immediately after centrifugation. Water volume was calculated using the formula: V = [(FW − ΔW)/ρH_2_O]/FW. FW = fresh weight of the plant, ΔW = weight of the plant after centrifugation, ρH_2_O = water density.

For the measurement of the apoplastic air volume, control and treated plants were collected, weighed, and put into pycnometers with distilled water. The pycnometer containing the plant and water was weighed and subjected to a vacuum. After the air was removed, the pycnometer was refilled with distilled water and weighed again. Air volume was calculated using the formula: V = [(Wa − Wb)ρH_2_O]/FW. Wa = weight of the pycnometer containing the plant and water after vacuum infiltration, Wb = weight of the pycnometer including the plant and water before infiltration, FW = fresh weight of plant, ρH_2_O = water density.

## References

[1] Bailey-Serres J., Fukao T., Gibbs D.J., Holdsworth M.J., Lee S.C., Licausi F., Perata P., Voesenek L.A., van Dongen J.T. (2012). Making sense of low oxygen sensing. Trends Plant Sci..

[2] Bailey-Serres J., Voesenek L.A.C.J. (2008). Flooding stress, acclimations and genetic diversity. Annu. Rev. Plant Biol..

[3] Gibbs D.J., Lee S.C., Isa N.M., Gramuglia S., Fukao T., Bassel G.W., Correia C.S., Corbineau F., Theodoulou F.L., Bailey-Serres J. (2011). Homeostatic response to hypoxia is regulated by the N-end rule pathway in plants. Nature.

[4] Licausi F., Kosmacz M., Weits D.A., Giuntoli B., Giorgi F.M., Voesenek L.A.C.J., Perata P., van Dongen J.T. (2011). Oxygen sensing in plants is mediated by an N-end rule pathway for protein destabilization. Nature.

[5] Voesenek L.A., Bailey-Serres J. (2015). Flood adaptive traits and processes, an overview. New Phytol..

[6] Wang F., Chen Z.H., Shabala S. (2017). Hypoxia Sensing in Plants, On a Quest for Ion Channels as Putative Oxygen Sensors. Plant Cell Physiol..

[7] Schmidt R.R., Fulda M., Paul M.V., Anders M., Plum F., Weits D.A., Kosmacz M., Larson T.R., Graham I.A., Beemster G.T.S. (2018). Low-oxygen response is triggered by an ATP-dependent shift in oleoyl-CoA in Arabidopsis. Proc. Natl. Acad. Sci. USA.

[8] Zhou Y., Tan W.J., Xie L.J., Qi H., Yang Y.C., Huang L.P., Lai Y.X., Tan Y.F., Zhou D.M., Yu L.J. (2019). Polyunsaturated linolenoyl-CoA modulates ERF-VII-mediated hypoxia signaling in Arabidopsis. J. Integr. Plant Biol..

[9] Shockey J.M., Fulda M.S., Browse J.A. (2002). Arabidopsis contains nine long-chain acyl-coenzyme a synthetase genes that participate in fatty acid and glycerolipid metabolism. Plant Physiol..

[10] Fulda M., Shockey J., Weber M., Wolter F.P., Heinz E. (2002). Two longchain acyl-CoA synthetases from Arabidopsis thaliana involved in peroxisomal fatty acid b-oxidation. Plant J..

[11] Schnurr J., Shockey J., Browse J. (2004). The acyl-CoA synthetase encoded by LACS2 is essential for normal cuticle development in Arabidopsis. Plant Cell.

[12] Bessire M., Chassot C., Jacquat A.C., Humphry M., Borel S., Petétot J.M., Métraux J.P., Nawrath C. (2007). A permeable cuticle in Arabidopsis leads to a strong resistance to Botrytis cinerea. EMBO J..

[13] Lü S., Song T., Kosma D.K., Parsons E.P., Rowland O., Jenks M.A. (2009). Arabidopsis CER8 encodes LONG-CHAIN ACYL-COA SYNTHETASE 1 (LACS1) that has overlapping functions with LACS2 in plant wax and cutin synthesis. Plant J..

[14] Weng H., Molina I., Shockey J., Browse J. (2010). Organ fusion and defective cuticle function in a lacs1 lacs2 double mutant of Arabidopsis. Planta.

[15] Zhao L., Katavic V., Li F., Haughn G.W., Kunst L. (2010). Insertional mutant analysis reveals that long-chain acyl-CoA synthetase 1 (LACS1), but not LACS8, functionally overlaps with LACS9 in Arabidopsis seed oil biosynthesis. Plant J..

[16] Jessen D., Olbrich A., Knüfer J., Krüger A., Hoppert M., Polle A., Fulda M. (2011). Combined activity of LACS1 and LACS4 is required for proper pollen coat formation in Arabidopsis. Plant J..

[17] Tang  D., Simonich M.T., Innes R.W. (2007). Mutations in LACS2, a long-chain acyl-coenzyme A synthetase, enhance susceptibility to avirulent Pseudomonas syringae but confer resistance to Botrytis cinerea in Arabidopsis. Plant Physiol..

[18] Xie L.J., Chen Q.F., Chen M.X., Yu L.J., Huang L., Chen L., Wang F.Z., Xia F.N., Zhu T.R., Wu J.X. (2015). Unsaturation of very-long-chain ceramides protects plant from hypoxia-induced damages by modulating ethylene signaling in Arabidopsis. PLoS Genet..

[19] Kurokawa Y., Nagai K., Huan P.D., Shimazaki K., Qu H., Mori Y., Toda Y., Kuroha T., Hayashi N., Aiga S. (2018). Rice leaf hydrophobicity and gas films are conferred by a wax synthesis gene (LGF1) and contribute to flood tolerance. New Phytol..

[20] Van den Dries N., Giannì S., Czerednik A., Krens F.A., de Klerk G.J. (2013). Flooding of the apoplast is a key factor in the development of hyperhydricity. J. Exp. Bot..

[21] Pollard M., Beisson F., Li Y.H., Ohlrogge J.B. (2008). Building lipid barriers, Biosynthesis of cutin and suberin. Trends Plant Sci..

[22] Kunst L., Samuels L. (2009). Plant cuticles shine, advances in wax biosynthesis and export. Curr. Opin. Plant Biol..

[23] Beisson F., Li-Beisson Y., Pollard M. (2012). Solving the puzzles of cutin and suberin polymer biosynthesis. Curr. Opin. Plant Biol..

[24] Bernard A., Joubès J. (2013). Arabidopsis cuticular waxes, advances in synthesis, export and regulation. Prog. Lipid Res..

[25] Yeats T.H., Rose J.K. (2013). The formation and function of plant cuticles. Plant Physiol..

[26] Li-Beisson Y., Shorrosh B., Beisson F., Andersson M.X., Arondel V., Bates P.D., Baud S., Bird D., Debono A., Durrett T.P. (2013). Acyl-lipid metabolism. Arab. Book.

[27] Xia Y., Yu K., Gao Q.M., Wilson E.V., Navarre D., Kachroo P., Kachroo A. (2012). Acyl-CoA Binding Proteins are required for cuticle formation and plant responses to microbes. Front. Plant Sci..

[28] Xue Y., Xiao S., Kim J., Lung S.C., Chen L., Tanner J.A., Suh M.C., Chye M.L. (2014). Arabidopsis membrane-associated acyl-CoA-binding protein ACBP1 is involved in stem cuticle formation. J. Exp. Bot..

[29] Xiao G.H., Wang K., Huang G., Zhu Y.X. (2016). Genome-scale analysis of the cotton *KCS* gene family revealed a binary mode of action for gibberellin A regulated fiber growth. J. Integr. Plant Biol..

[30] Kim H., Choi D., Suh M.C. (2017). Cuticle ultrastructure, cuticular lipid composition, and gene expression in hypoxia-stressed Arabidopsis stems and leaves. Plant Cell Rep..

[31] Boyer J.S., Wong S.C., Farquhar G.D. (1997). CO_2_ and water vapor exchange across leaf cuticle (epidermis) at various water potentials. Plant Physiol..

[32] Frost-Christensen H., Jørgensen L.B., Floto F. (2003). Species specificity of resistance to oxygen diffusion in thin cuticular membranes from amphibious plants. Plant Cell Environ..

[33] Mommer L., Pons T.L., Wolters-Arts M., Venema J.H., Visser E.J. (2005). Submergence-induced morphological, anatomical, and biochemical responses in a terrestrial species affect gas diffusion resistance and photosynthetic performance. Plant Physiol..

[34] Kannangara R., Branigan C., Liu Y., Penfield T., Rao V., Mouille G., Höfte H., Pauly M., Riechmann J.L., Broun P. (2007). The Transcription Factor WIN1/SHN1 Regulates Cutin Biosynthesis in Arabidopsis thaliana. Plant Cell.

[35] Xie L.J., Yu L.J., Chen Q.F., Wang F.Z., Huang L., Xia F.N., Zhu T.R., Wu J.X., Yin J., Liao B. (2015). Arabidopsis acyl-CoA-binding protein ACBP3 participates in plant response to hypoxia by modulating very-long-chain fatty acid metabolism. Plant J..

[36] Peng X., Wang M., Li Y., Yan W., Chang Z., Chen Z., Xu C., Yang C., Deng. X.W., Wu J. (2019). Lectin receptor kinase OsLecRK-S.7 is required for pollen development and male fertility. J. Integr. Plant Biol..

[37] Koch K., Barthlott W. (2009). Superhydrophobic and superhydrophilic plant surfaces, an inspiration for biomimetic materials. Philos. Trans. R. Soc. A Math. Phys. Eng. Sci..

[38] Lee S.B., Go Y.S., Bae H.J., Park J.H., Cho S.H., Cho H.J., Lee D.S., Park O.K., Hwang I., Suh M.C. (2009). Disruption of glycosylphosphatidylinositol-anchored lipid transfer protein gene altered cuticular lipid composition, increased plastoglobules, and enhanced susceptibility to infection by the fungal pathogen Alternaria brassicicola. Plant Physiol..

[39] Tanaka T., Tanaka H., Machida C., Watanabe M., Machida Y. (2004). A new method for rapid visualization of defects in leaf cuticle reveals five intrinsic patterns of surface defects in Arabidopsis. Plant J..

[40] Chen L., Liao B., Qi H., Xie L.J., Huang L., Tan W.J., Zhai N., Yuan L.B., Zhou Y., Yu L.J. (2015). Autophagy contributes to regulation of the hypoxia response during submergence in Arabidopsis thaliana. Autophagy.

[41] Lee S.H., Sakuraba Y., Lee T., Kim K.W., An G., Lee H.Y., Paek N.C. (2015). Mutation of Oryza sativa CORONATINE INSENSITIVE 1b (OsCOI1b) delays leaf senescence. J. Integr. Plant Biol..

[42] Terry M.E., Bonner B.A. (1980). An examination of centrifugation as a method of extracting an extracellular solution from peas, and its use for the study of indoleacetic acid-induced growth. Plant Physiol..

[43] Raskin I. (1983). A method for measuring leaf volume, density, thickness, and internal gas volume. HortScience.

